# Supplementary material for: Gene-drive suppression of mosquito populations in large cages as a bridge between lab and field
Source: Nat Commun. 2021 Jul 28;12:4589. doi: 10.1038/s41467-021-24790-6 (PMC8319305; doi:10.1038/s41467-021-24790-6)
Supplement: Supplementary file 1 — Supplementary Information [file 41467_2021_24790_MOESM1_ESM.pdf]

Model description and fitting method description

Full model description

Model summary

We simulated the experiment using an individual based stochastic model in which mosquitoes are grouped by life-stage (larvae or adult), age, sex, and genotype. Adult females are further grouped by whether or not they have mated (it is assumed that females mate only once) and, if they have, by the genotype of their mate. Finally, females are grouped by whether either parent, or both parents, carried the drive allele. We assume there are three possible allele types at the dsx locus: wildtype (W), Ag(QFS)1 drive construct (D), and non-functional alleles that are resistant to homing (R).

The model simulates the daily changes to the population that result from egg laying, deaths, and matings. Each of these processes is stochastic, with probabilities that depend on the mosquito groupings. For example, the per day survival probability of adult mosquitoes reduces with age, while the egg production of a female will depend on her genotype and possibly her parental genotypes. The model replicates the experimental design with respect to twice-weekly egg-laying, the initiation phase, the transgene introductions, and the subsequent monitoring phase. The model parameters and their default values are listed in Supplementary Table 1.

Egg laying

Mated females lay eggs twice each week (on Mondays and Thursdays), and the probability a given female lays is p_{Lay}. For those that do, the number of eggs is Poisson distributed with expectation $\theta \times \omega_i^q$ where θ is the fertility of wildtype females, and ω_i^q is the relative fertility of females whose genotype is i and the superscript q is either f, m, n or b to indicate, respectively, that the mother had the drive allele, the father had it, neither had it, or both had it. We assume females need at least one copy of the wildtype allele to produce eggs, and so $\omega_{DD}^q = \omega_{DR}^q = \omega_{RR}^q = 0 \quad \forall q$. We set the maternal and paternal drive allele fitness costs to be ρ_f and ρ_m. This gives

$$\begin{aligned}
\omega_{WW}^n &= \omega_{WR}^n = 1 \\
\omega_{WW}^f &= \omega_{WR}^f = 1 - \rho_f \\
\omega_{WW}^m &= \omega_{WR}^m = 1 - \rho_m \\
\omega_{WW}^b &= \omega_{WR}^b = (1 - \rho_f)(1 - \rho_m) \\
\omega_{WD}^f &= (1 - \rho_f)
\end{aligned}$$

$$\begin{aligned}\omega^m_{WD} &= (1-\rho_m)\\ \omega^b_{WD} &= (1-\rho_f)(1-\rho_m)\end{aligned}$$

The genotype of each egg is randomised depending on the parental genotypes. The inheritance probabilities are Mendelian except in the case of W/D parents for which we assume sex-specific homing rates as measured in small cages. Non-homed gametes from W/D individuals become R alleles with probability γ. A random sample of 400 larvae (or all the larvae if there are less than 400) are kept from each twice-weekly batch of eggs and added to the adult population after their development which takes ten days.

Deaths

We used the cage survival assays that were performed both before and after the population dynamic experiments to parameterise adult survival, as follows. For both the start and end survival assay, we fitted Weibull distributions to the survivorship curves separately for females and males. For each sex, we transformed the Weibull parameters linearly from the start to the end of the experiment. On day d, we thus have

$$\begin{aligned}k_i(d) &= k_i(0) + d \times \frac{k_i(T) - k_i(0)}{T},\\ \lambda_i(d) &= \lambda_i(0) + d \times \frac{\lambda_i(T) - \lambda_i(0)}{T}.\end{aligned}$$

The mortality probability of an adult dying on day d, $\mu_i(d,a)$, is then determined by a function that depends on d as well as the individual's age, a, and sex, $i \in \{m, f\}$. This has the form:

$$\mu_i(d,a) = 1 - e^{\left(\frac{a}{\lambda_i(d)}\right)^{k_i(d)}} - e^{\left(\frac{1+a}{\lambda_i(d)}\right)^{k_i(d)}}$$

Mating

A virgin female will mate on a given day with a probability $\frac{M_{\text{Tot}}}{M_{\text{Tot}}+\beta}$ where M_{Tot} is the total number of adult males and β is a parameter that controls the extent to which mating becomes limited when there are few males in the population. We set $\beta = 100$, which means that most virgin females will mate on the first day after their emergence (mating probability $> 1/2$ per day if there are > 100 males in the cage). The male a female mates with is randomised from all the males in the cage (neither genotype nor age affects mating ability).

ABC Model fitting

We attempt to fit five model parameters from the model to data comparison: $\rho_f, \rho_m, \gamma, p_{\text{Lay}}$, and θ.

Parameter	interpretation	default value	source
e_m	Homing rate in males	0.9635	[2]
e_f	Homing rate in females	0.9985	[2]
β	Number of males when females mate with probability 1/2 per day	100	Expert judgement
$(k_f(0), k_f(T), k_m(0), k_m(T))$	Weibull shape parameters, for females $\cdot_f$ and males $\cdot_m$, at the start (0) or end (T) of the experiment	$(2.33, 2.39, 2.69, 1.79)$	Survival assays
$(\lambda_f(0), \lambda_f(T), \lambda_m(0), \lambda_m(T))$	Weibull scale parameters, for females $\cdot_f$ and males $\cdot_m$, at the start (0) or end (T) of the experiment	$(6.50, 15.91, 5.21, 10.38)$	Survival assays
ρ_f	Fitness cost of paternal cas9 deposition	$0.67^{*\dagger}$	[2]
ρ_m	Fitness cost of maternal cas9 deposition	$0^{*\dagger}$	[2]
γ	Proportion of non-homed alleles that form R alleles	0.5	
p_{Lay}	Proportion of females that lay eggs at a given opportunity	0.1^{*}	[1]
θ	Number of eggs per batch from a fully fit female	100^{*}	Expert judgement

Supplementary Table 1: Model parameters. *: we also inferred this parameter from the cage data; †: the default values for these parameters are derived from the fertility of heterozygous females as measured by [2] for the different parental combinations of genotypes.

Fitting method

We fit the these parameters using a simple Monte-Carlo algorithm based on 'approximate Bayesian computation' (ABC). In short:

1. Parameters are drawn at random from a prior distribution. The parameter vector is called a *particle*.
2. A number of simulations are run with this particle.
3. The simulations are scored for their closeness to the cage data according to a number of distance measures (below).
4. Steps 1-3 are repeated lots of times.
5. The particles are filtered using the distance scores. E.g. we retain all particles where each distance score is in the lowest q quantile (for some q).
6. The retained particles are the posterior distribution.

Distance metrics

Time-series smoothing: transgene frequency and male ratio We first smooth each time-series in the data $\{a_d\}_{d=1..T}$, where T is the length of the time series, by transfroming it into a 2 week moving average time-series $\{S(a)_d\}_{d=3..T-2}$. Note that the smoothed time series is four data points fewer than the original series because there are two data points per week each original series. We compute the sum of square differences between a_d and $S(a)$, $R(a) = \sum_{d=3}^{T-2}(a_d - S(a)_d)^2$. We apply the same smoothing and residual functions to the simulated data, to get corresponding variables $S(b)$ and $R(b)$. By applying these transformations to the two sets of treatment time series, we obtain four distance measures between data and simulated data.

1. Transgene frequency trend distance. This distance, d_1, is the sum of square differences between all corresponding pairs of real and simulated transgene frequency time-series:

$$d_1 = \sum_{i=1,2}\sum_{j=1,2}\sum_{k=1}^{N_{sim}}\left(\sum_d (S(a)_d^{(i,j)} - S(b)_d^{(i,k)})^2\right) \quad (1)$$

   where i refers to the experimental treatment (25% or 50%) starting transgene frequency, j refers to the data replicate, k refers to the simulation replicate, and d refers to the day within the (smoothed) time series.

2. Transgene frequency noise distance. This distance, d_2, is the sum of square differences between all corresponding pairs of real and simulated transgene frequency residuals:

$$d_1 = \sum_{i=1,2}\sum_{j=1,2}\sum_{k=1}^{N_{sim}}\left((R(a)^{(i,j)} - R(b)^{(i,k)})^2\right) \quad (2)$$

3. Male and homozygous female proportion trend. This distance, d_3, is computed as d_1 but using the male and homozygous female time series.
4. Male and homozygous female proportion noise (d_4). As d_2 but again using the residuals of the male and homozygous female time series.

Control cage egg number In addition, we obtain two distance measures from the control cage egg number data. To do so we compute the mean and variance of the two time series of egg number in the control cages, from day -53 to $+248$. This gives $M(e^j) = \text{Mean}(e_d^j)$ and $V(e^j) = \text{Variance}(e_d^j)$ for replicate j (out of two replicates), and we obtain similarly $M(e_{sim}^k)$ and $V(e_{sim}^k)$ for the simulated control cages.

5. Egg number mean (d_5). This distance is $\sum_{j=1,2}\sum_{k=1}^{N_{sim}}\left(M(e^j) - M(e_{sim}^k)\right)^2$.
6. Egg number variance (d_6). This distance is $\sum_{j=1,2}\sum_{k=1}^{N_{sim}}\left(V(e^j) - V(e_{sim}^k)\right)^2$.

r2 allele data Finally, we obtain one distance measure, d_7, based on the observed number of r2 alleles at different time points. We denote the data point $c^{(i,j,d)}$ to be the observed frequency of r2 alleles based on $N^{(i,j,d)}$ samples, where i is treatment, j is replicate, and d is day. Similarly, $c_{sim}^{(i,k,d)}$ is the proportion of r2 alleles given in the kth replicate of the simulation of treatment i on day d.

We construct a likelihood function for observing $c^{(i,j,d)}$ if the simulation proportion of r2 alleles is correct:

$$like(c^{(i,j,d)}) = PDF[\text{Binomial distribution}(N^{(i,j,d)}, c_{sim}^{(i,k,d)}), N^{(i,j,d)} * c^{(i,j,d)})].$$

7. d_7 is then the sum of log-likelihoods over all data points: $d_7 = -1 * \sum_{i=1,2}\sum_{k=1}^{N_{sim}}\left(Log[like(c^{(i,j,d)})]\right)$. Note we multiply by minus one because smaller distances should correspond to a better correspondence of data and simulation.

References

[1] Andrew Hammond, Xenia Karlsson, Ioanna Morianou, Kyros Kyrou, Andrea Beaghton, Matthew Gribble, Nace Kranjc, Roberto Galizi, Austin Burt, Andrea Crisanti, et al. Regulating the expression of gene drives is key to increasing their invasive potential and the mitigation of resistance. *PLoS genetics*, 17(1):e1009321, 2021.

[2] Kyros Kyrou, Andrew M. Hammond, Roberto Galizi, Nace Kranjc, Austin Burt, Andrea K. Beaghton, Tony Nolan, and Andrea Crisanti. A CRISPR-Cas9 gene drive targeting doublesex causes complete population suppression in caged *Anopheles gambiae* mosquitoes. *Nat Biotechnol*, 36:1062, September 2018.

Model description and fitting method description

References

[1] Andrew M Hammond, Xenia Karlsson, Ioanna Morianou, Kyros Kyrou, Andrea Beaghton, Matthew Gribble, Nace Kranjc, Roberto Galizi, Austin Burt, Andrea Crisanti, et al. Regulation of gene drive expression increases invasive potential and mitigates resistance. *bioRxiv*, page 360339, 2020.

[2] Kyros Kyrou, Andrew M. Hammond, Roberto Galizi, Nace Kranjc, Austin Burt, Andrea K. Beaghton, Tony Nolan, and Andrea Crisanti. A CRISPR-Cas9 gene drive targeting doublesex causes complete population suppression in caged *Anopheles gambiae* mosquitoes. *Nat Biotechnol*, 36:1062, September 2018.

Supplementary Table 2. Posterior estimates of model parameters. The cost parameters reduce the fertility of females with a transgenic parent, that may or may not be transgenic themselves. The eggs per batch parameter is the estimated batch size of females that neither carry the transgene, nor had a transgenic parent. Laying probability is assumed to affect all females equally. The NHEJ rate is the probability that a non-transgenic gamete produced by a transgene-heterozygous parent is a non-functional R2 allele rather than a wildtype allele.

		Posterior			
Parameter	Prior	Mean	2.5%	50%	97.5%
Maternal deposition cost	U(0,1)	0.46	0.015	0.46	0.96
Paternal deposition cost	U(0,1)	0.65	0.42	0.66	0.82
Eggs per batch	U(50,250)	116	51	112	213
Laying probability	U(0.05,0.5)	0.14	0.08	0.13	0.21
NHEJ rate	U(0,1)	0.50	0.27	0.49	0.83

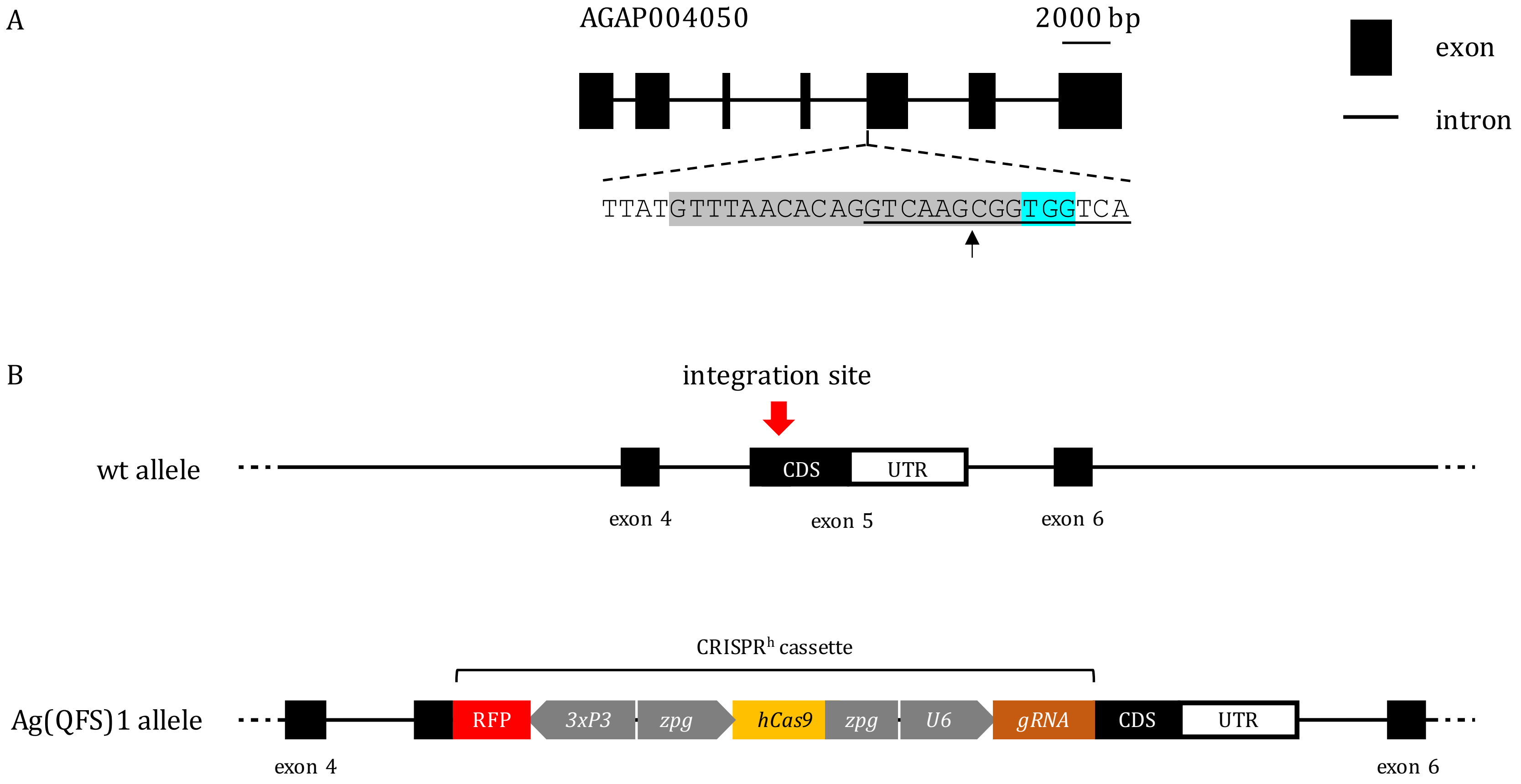

A
AGAP004050
2000 bp
exon
intron
TTATGTTTAACACAGGTCAAGCGGTGGTCA
B
integration site
wt allele
CDS
UTR
exon 4
exon 5
exon 6
CRISPR^h cassette
Ag(QFS)1 allele
RFP
3xP3
zpg
hCas9
zpg
U6
gRNA
CDS
UTR
exon 4
exon 6

Supplementary Figure 1. The gRNA sequence and the integration site of Ag(QFS)1. (A) The gRNA used in Ag(QFS)1 to target the female-specific exon of the *dsx* gene. The gRNA overlaps the intron 4/ exon 5 boundary of the gene, inducing a double-strand break at the start of the exon (arrow). The gRNA sequence is shaded in grey and the PAM sequence in blue. The underlined section indicates the sequence of the female-specific exon 5. Introns are not drawn to scale. (B) The $dsxF^{CRISPRh}$ construct integrated within the genome of the Ag(QFS)1 strain. The $CRISPR^{h}$ cassette is integrated within the coding sequence of the female-specific exon 5 of the *dsx* gene in *Anopheles gambiae* (arrow), disrupting the function of the exon and the female-specific isoform of the gene. The cassette carries a human codon-optimised Cas9 gene under the regulation of the *zpg* regulatory elements, the exon-5-specific gRNA shown in (A) under the ubiquitously expressed U6 promoter, and a dsRed fluorescent protein marker (RFP). Elements are not drawn to scale.

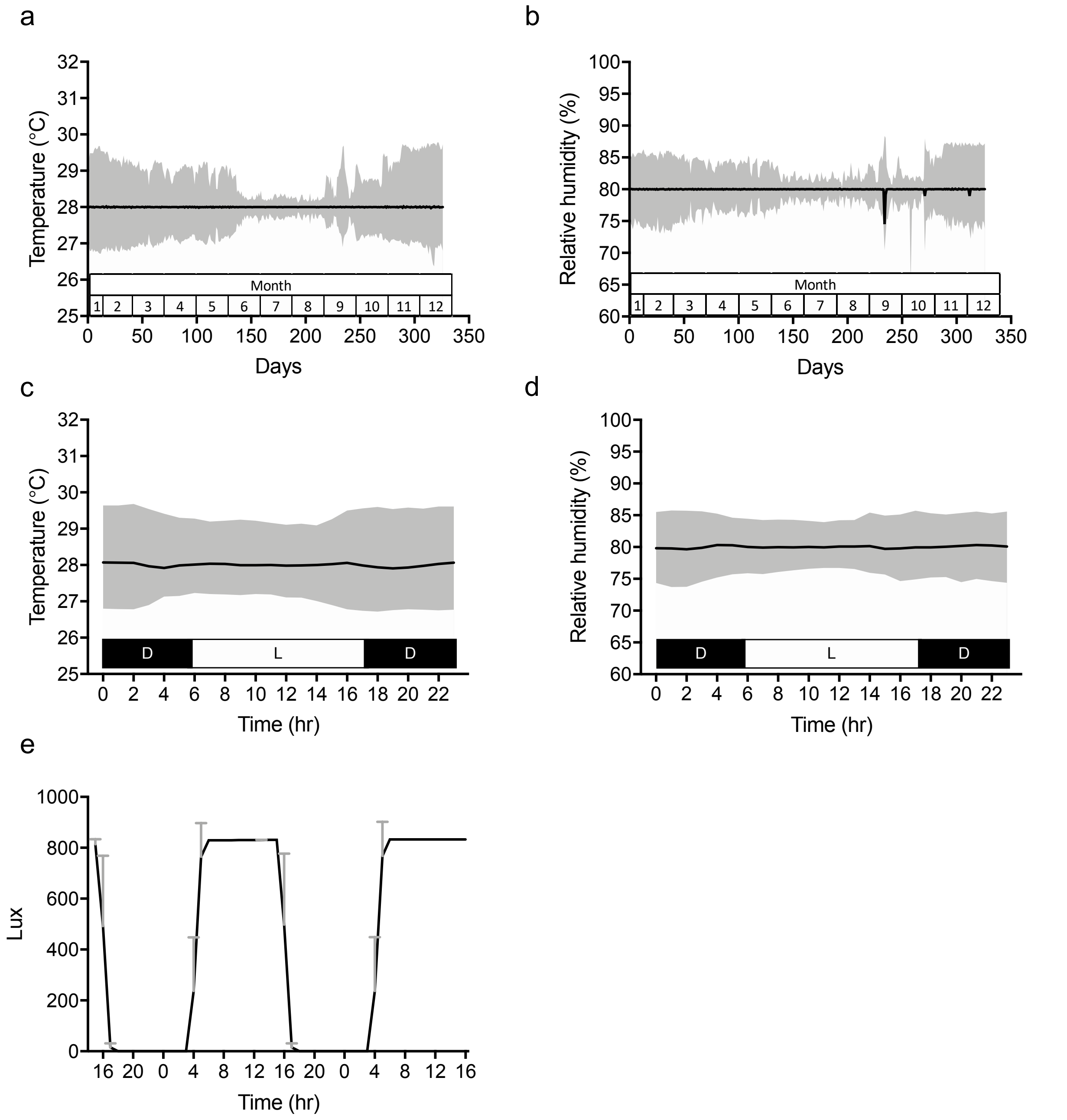

a
b
c
d
e
Temperature (°C)
Relative humidity (%)
Days
Month
1
2
3
4
5
6
7
8
9
10
11
12
D
L
D
Time (hr)
Lux

Supplementary Fig 2. Environmental conditions recorded during the course of the experiment in the large cages. Mean daily and hourly temperature (a, c) and relative humidity (b, d) recorded on a 1-minute interval (solid black line, n=1440) with upper and lower data recorded (grey shading bands) show a very stable environment of 28°C (± 1.5) and 80% (± 5) relative humidity. Recordings took place during the period 21/01/2019 to 23/12/2019, with the month of the year indicated in boxes next to the time axis. A lower variation of min-max values for both temperature and humidity was observed during the summer months (June-August). No day-night variation in mean temperature or humidity was recorded (c, d). Grey shading bands represent upper and lower data recorded (n=326). e) Hourly mean light intensity (in lux) recorded next to the bricks shelter from cage 3 (in the middle of the chamber) over 2 days with a 1-minute interval. Error bars indicate standard deviation (n=60).

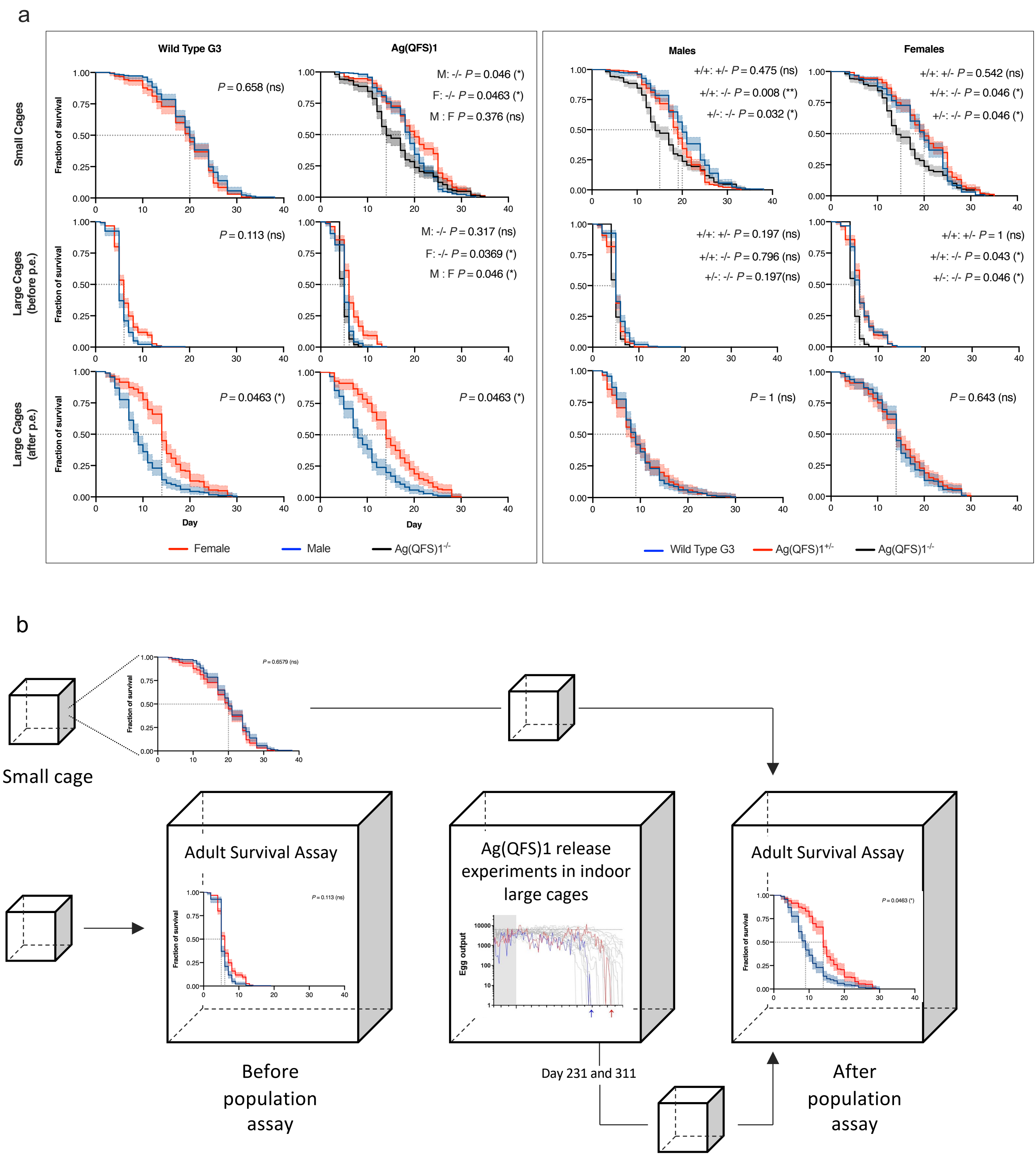
a
Wild Type G3
Ag(QFS)1
Males
Females
Small Cages
Large Cages (before p.e.)
Large Cages (after p.e.)
Fraction of survival
Day
P = 0.658 (ns)
M: -/- P = 0.046 (*)
F: -/- P = 0.0463 (*)
M : F P = 0.376 (ns)
+/+: +/- P = 0.475 (ns)
+/+: -/- P = 0.008 (**)
+/-: -/- P = 0.032 (*)
+/+: +/- P = 0.542 (ns)
+/+: -/- P = 0.046 (*)
+/-: -/- P = 0.046 (*)
P = 0.113 (ns)
M: -/- P = 0.317 (ns)
F: -/- P = 0.0369 (*)
M : F P = 0.046 (*)
+/+: +/- P = 0.197 (ns)
+/+: -/- P = 0.796 (ns)
+/-: -/- P = 0.197(ns)
+/+: +/- P = 1 (ns)
+/+: -/- P = 0.043 (*)
+/-: -/- P = 0.046 (*)
P = 0.0463 (*)
P = 0.0463 (*)
P = 1 (ns)
P = 0.643 (ns)
Female
Male
Ag(QFS)1-/-
Wild Type G3
Ag(QFS)1+/-
Ag(QFS)1-/-
b
Small cage
Adult Survival Assay
Ag(QFS)1 release experiments in indoor large cages
Adult Survival Assay
Egg output
Day 231 and 311
Before population assay
After population assay

Supplementary Figure 3. Wild-type G3 and Ag(QFS)1 adult survival curves in small and large cages. a) Wild-type G3 and Ag(QFS)1 adults were monitored for daily survival in small cages, and large cages assessed before and after the population experiments (p.e.). Statistical difference of the median survival (grey dotted lines) using the Kruskal-Wallis nonparametric test (one-sided, n=3, df=1) was used to compared between male and female of same genotype (left-hand panels) or between genotypes within same gender (right-hand panels). Ag(QFS)1 homozygotes males and females were also monitored in small cages and large cages before the population experiments were conducted. Since homozygous individuals were tested unsexed because females exhibit male morphological traits, they are indicated as black line irrespective of the sex. Error bands (coloured shadings) represents the 95% Confidence Intervals of the mean. Experiments were performed with three independent biological replicates. b) Overview of the time point for assessment of adult survival in small and large cages (represented as small and large cubes, respectively) in respect to the release experiment in large cages.

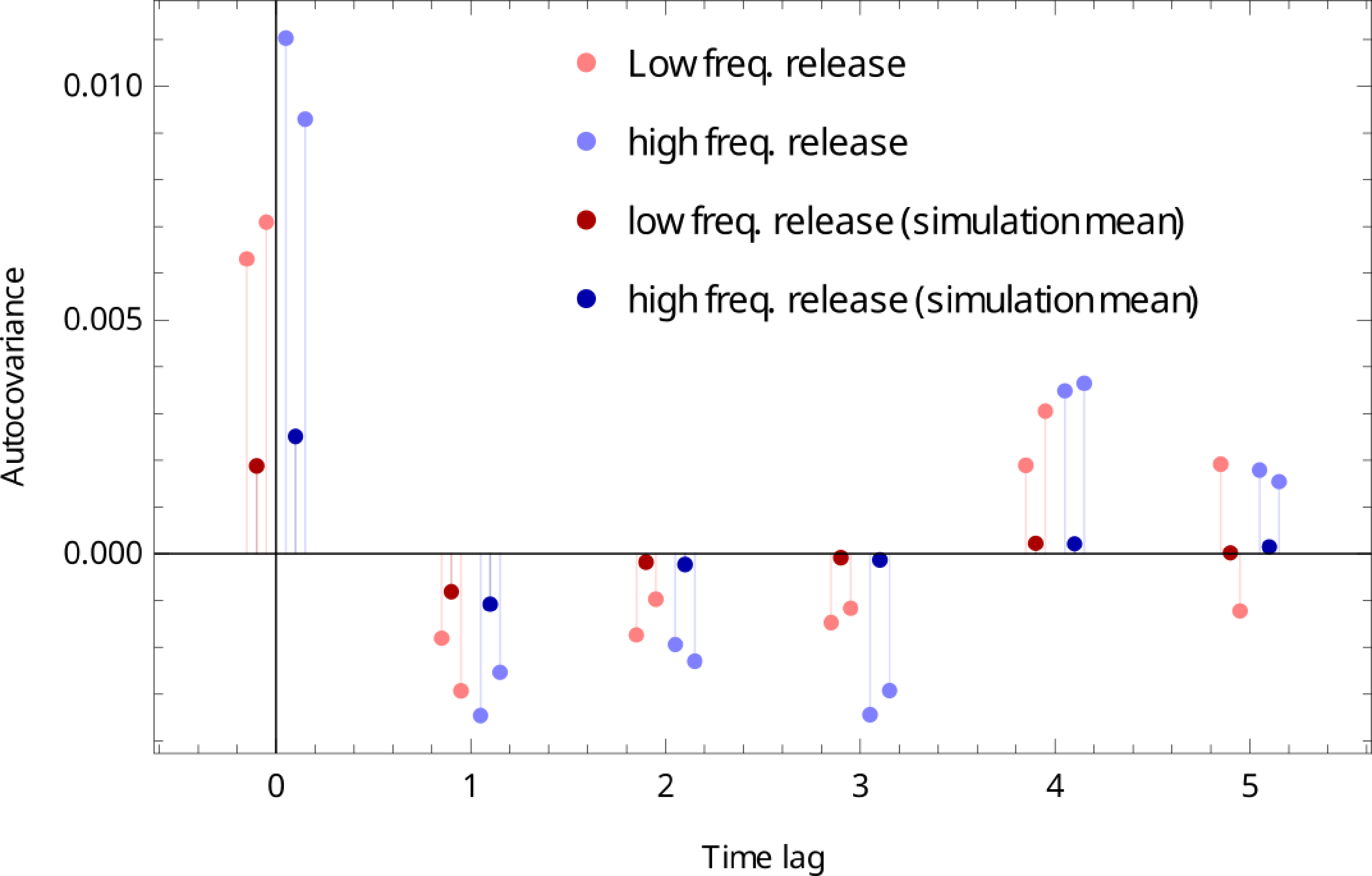

Low freq. release
high freq. release
low freq. release (simulation mean)
high freq. release (simulation mean)
Autocovariance
0.010
0.005
0.000
0
1
2
3
4
5
Time lag

Supplementary Figure 4. Temporal autocovariance in the gene drive allelic frequency time series. For each time series, a ‘residual’ series without trend was computed by subtracting a lag 1 moving average of the time series from itself. Autocovariance of the residual series was then calculated at time lags up to 5 half-weeks (the drive allelic frequency was observed twice per week). For comparison, the darker color points show the means of applying the same calculation to 100 simulations using default parameters (Supp. Methods).

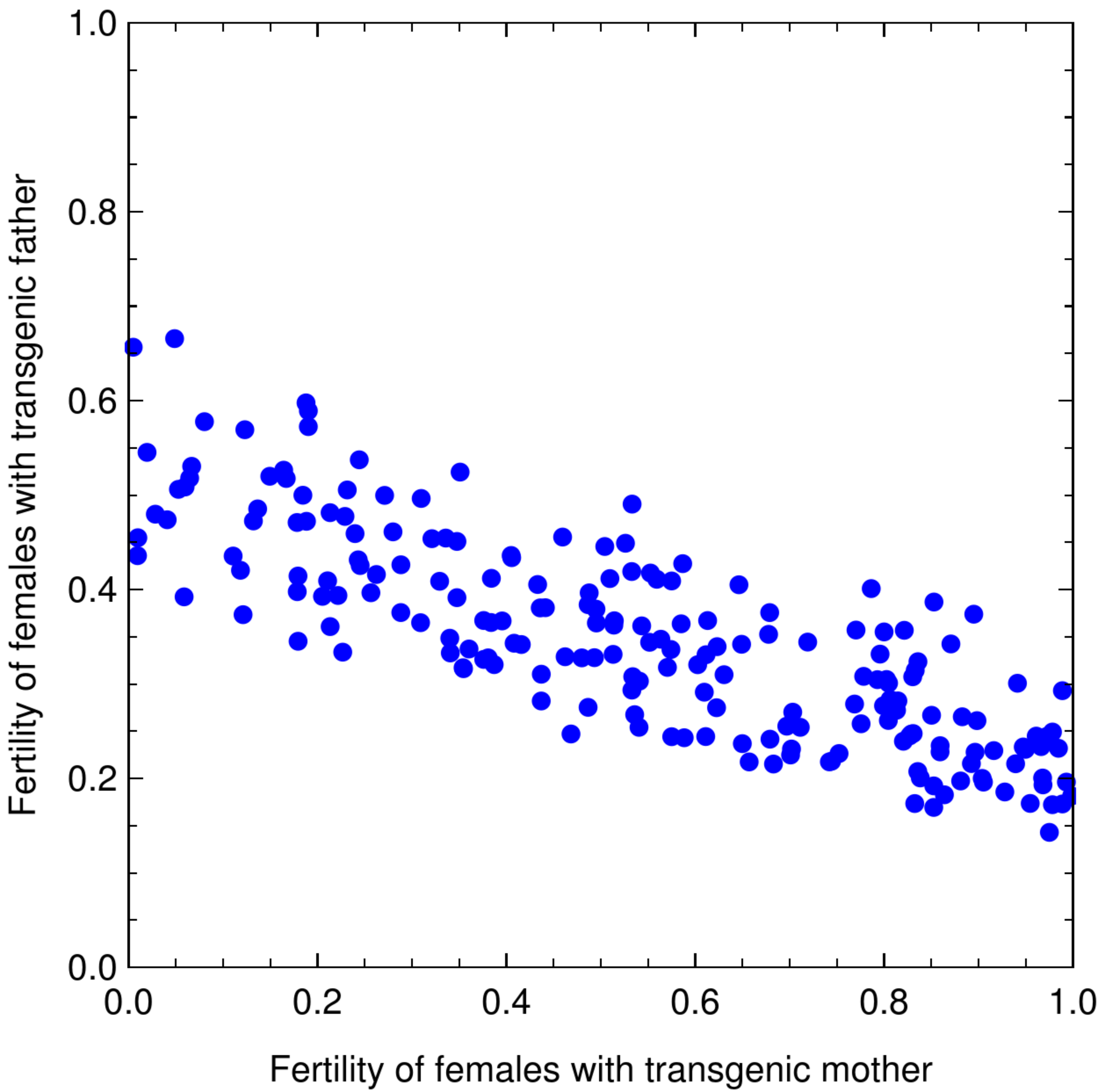

1.0
0.8
0.6
0.4
0.2
0.0
Fertility of females with transgenic father
0.0
0.2
0.4
0.6
0.8
1.0
Fertility of females with transgenic mother

Supplementary Figure 5. Posterior distribution of female fertility parameters. Based on 50,000 samples of the prior distribution (each simulated 10 times), filtered to 200 posterior points selected as particles in the lowest 26.17% quantile from all the distance measures described (Supp. Methods) except d6 (Egg number variance), which proved unreliable because the cage variability in egg number cannot be replicated by the model. The prior distribution is uniform across the unit square.

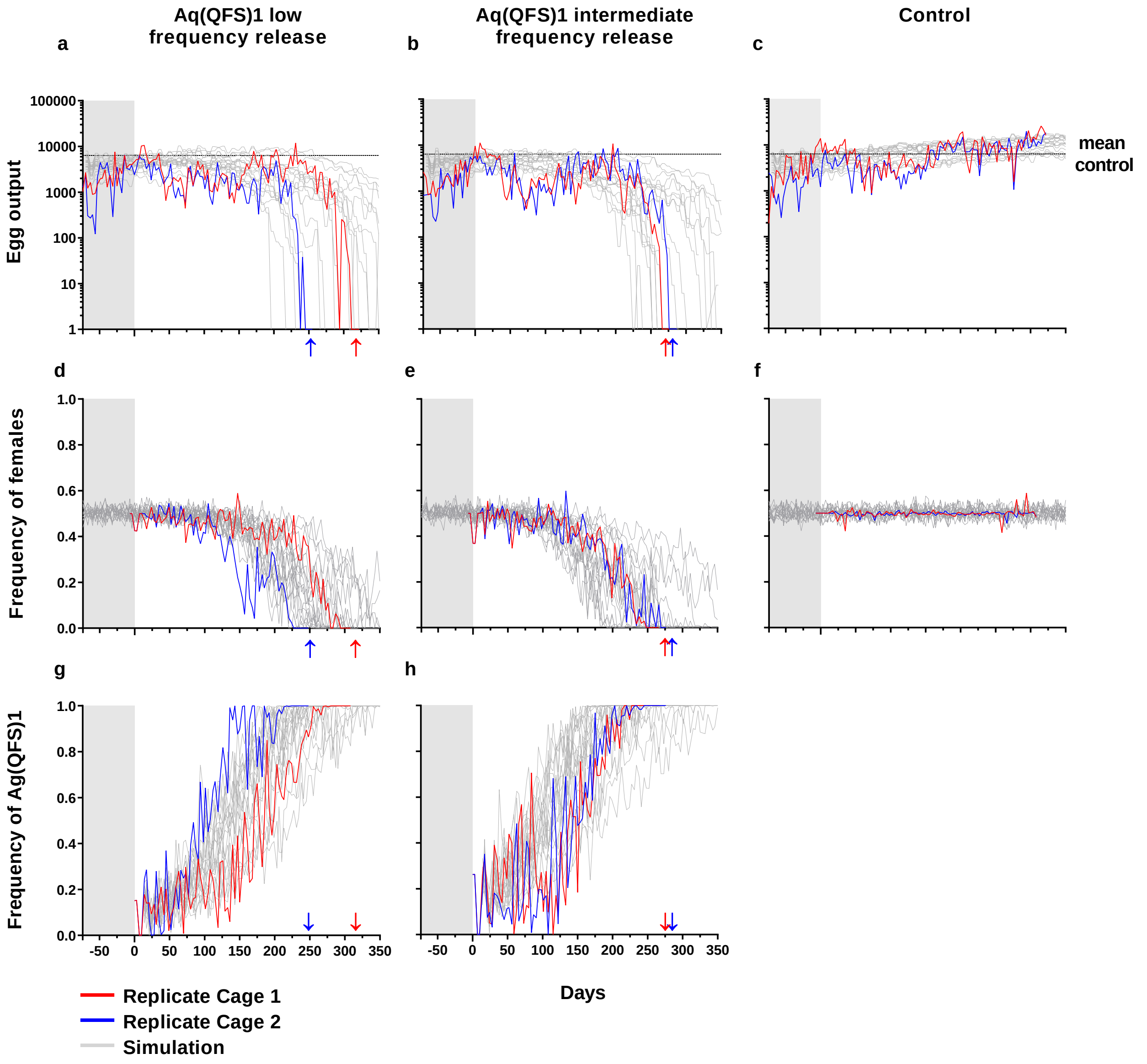
Aq(QFS)1 low frequency release
Aq(QFS)1 intermediate frequency release
Control
a
b
c
Egg output
100000
10000
1000
100
10
1
mean control
d
e
f
Frequency of females
1.0
0.8
0.6
0.4
0.2
0.0
g
h
Frequency of Ag(QFS)1
-50
0
50
100
150
200
250
300
350
Days
Replicate Cage 1
Replicate Cage 2
Simulation

Supplementary Figure 6. Comparison of model simulations (grey) and data (coloured) for the model with default parameters. Age-structured large cage (ASL) populations were established over a period of 74 days (shaded grey) and seeded in duplicate with Ag(QFS)1 heterozygous males at low (12.5%, panels a, d, g) and medium (25%, panels b, e, h) allelic frequency, whereas two control cages were maintained without introduction of the Ag(QFS)1 gene drive (c, f). The frequency of Ag(QFS)1 alleles (g, h), total egg output (a, b, c), and the frequency of non-intersex females (d, e, f) were monitored over time (red and blue lines for replicate cages). Arrows indicate the point at which no further eggs were recovered, the point at which populations were considered eliminated. A total of 20 stochastic simulations of the egg output and the frequency of AgQFS(1) (grey lines) were modelled using parameters drawn at random from the posterior distribution and superimposed to experimental data for the control and gene drive releases (a, b, c, g, h). Mean egg output of the control is indicated by a dashed line (a, b, c).

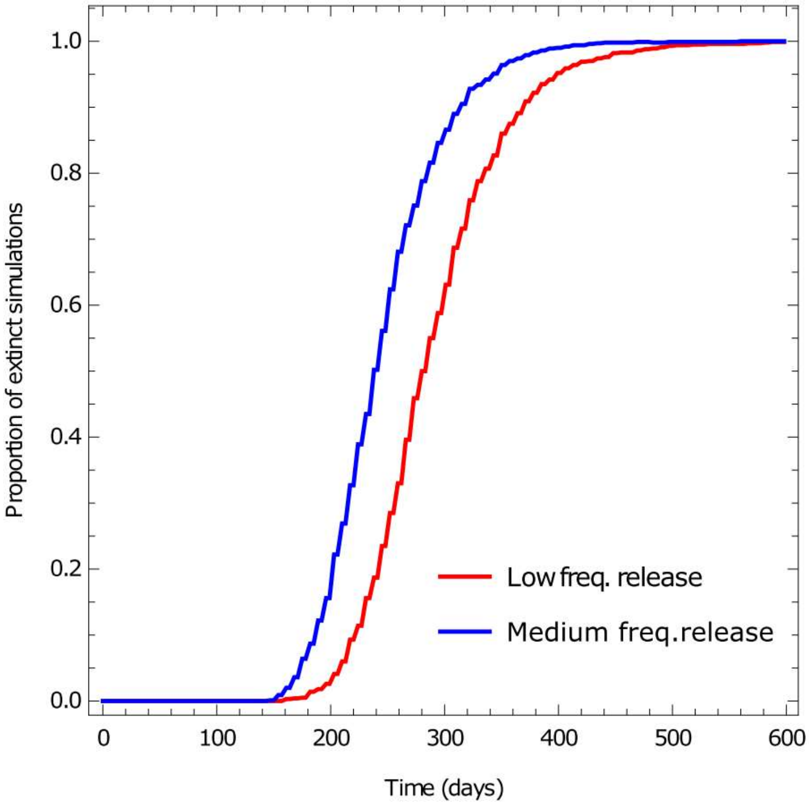

Proportion of extinct simulations
1.0
0.8
0.6
0.4
0.2
0.0
0
100
200
300
400
500
600
Time (days)
Low freq. release
Medium freq.release

Supplementary Figure 7. Stochastic simulations predict the likelihood of full suppression within 600 days. A total of 1000 stochastic simulations were performed for low (12.5%, red) and medium (25%, blue) allelic frequency releases of Ag(QFS)1 using parameters drawn at random from the posterior distribution. Plotted are the cumulative fraction of fully suppressed populations. Full suppression is predicted to occur within 500 days in 993 and 999 of 1000 simulations for the low (red) and medium (blue) frequency releases respectively, and in all simulations by 600 days.
