# Supplementary material for: Gene-drive suppression of mosquito populations in large cages as a bridge between lab and field
Source: Nat Commun. 2021 Jul 28;12:4589. doi: 10.1038/s41467-021-24790-6 (PMC8319305; doi:10.1038/s41467-021-24790-6)
Supplement: Supplementary file 3 — Reporting Summary [file 41467_2021_24790_MOESM3_ESM.pdf]

nature portfolio

Corresponding author(s): Ruth Müller

Last updated by author(s): 22-06-2021

Reporting Summary

Nature Portfolio wishes to improve the reproducibility of the work that we publish. This form provides structure for consistency and transparency in reporting. For further information on Nature Portfolio policies, see our Editorial Policies and the Editorial Policy Checklist.

Statistics

For all statistical analyses, confirm that the following items are present in the figure legend, table legend, main text, or Methods section.

n/a	Confirmed	
☐	☒	The exact sample size (n) for each experimental group/condition, given as a discrete number and unit of measurement
☐	☒	A statement on whether measurements were taken from distinct samples or whether the same sample was measured repeatedly
☐	☒	The statistical test(s) used AND whether they are one- or two-sided<br>*Only common tests should be described solely by name; describe more complex techniques in the Methods section.*
☐	☒	A description of all covariates tested
☐	☒	A description of any assumptions or corrections, such as tests of normality and adjustment for multiple comparisons
☐	☒	A full description of the statistical parameters including central tendency (e.g. means) or other basic estimates (e.g. regression coefficient) AND variation (e.g. standard deviation) or associated estimates of uncertainty (e.g. confidence intervals)
☐	☒	For null hypothesis testing, the test statistic (e.g. F, t, r) with confidence intervals, effect sizes, degrees of freedom and P value noted<br>*Give P values as exact values whenever suitable.*
☐	☒	For Bayesian analysis, information on the choice of priors and Markov chain Monte Carlo settings
☒	☐	For hierarchical and complex designs, identification of the appropriate level for tests and full reporting of outcomes
☒	☐	Estimates of effect sizes (e.g. Cohen's d, Pearson's r), indicating how they were calculated

Our web collection on statistics for biologists contains articles on many of the points above.

Software and code

Policy information about availability of computer code

Data collection	GraphPad Prism vs 9; Winkratos software (ANGELANTONI Industries S.p.A, Massa Martana, Italy); EggCounter v1.0 software; COPAS, Union Biometrica, Boston, USA; CRISPResso2 (Clement et al. 2019); R v3.4.0
Data analysis	GraphPad Prism vs 9; CRISPResso2 (Clement et al. 2019); R v3.4.0

For manuscripts utilizing custom algorithms or software that are central to the research but not yet described in published literature, software must be made available to editors and reviewers. We strongly encourage code deposition in a community repository (e.g. GitHub). See the Nature Portfolio guidelines for submitting code & software for further information.

Data

Policy information about availability of data

All manuscripts must include a data availability statement. This statement should provide the following information, where applicable:

- Accession codes, unique identifiers, or web links for publicly available datasets
- A description of any restrictions on data availability
- For clinical datasets or third party data, please ensure that the statement adheres to our policy

Data availability statement
The data generated in this study are provided as supplementary figures and tables and in the Supplementary Information/Source Data file.
>Supplementary Methods. Model description and fitting method description
>Supplementary Table 1. Posterior estimates of model parameters.
>Supplementary Figure 1. The gRNA sequence and the integration site of Ag(QFS)1.
>Supplementary Figure 2. Environmental conditions recorded during the course of the experiment in the large cages.

>Supplementary Figure 3. Wild-type G3 and Ag(QFS)1 adult survival curves in small and large cages.
>Supplementary Figure 4. Temporal autocovariance in the gene drive allelic frequency time series.
>Supplementary Figure 5. Posterior distribution of female fertility parameters.
>Supplementary Figure 6. Comparison of model simulations (grey) and data (coloured) for the model with default parameters.
>Supplementary Figure 7. Stochastic simulations predict the likelihood of full suppression within 600 days.
The raw data generated in this study are also available in the DRYAD database [https://doi.org/10.5061/dryad.9w0vt4bg0].
>Dataset 1: Ag(QFS)1 release experiments in large cages including Pooled amplicon sequencing and analysis.
>Dataset 2: Measured life history parameters including statistical analysis.
>Dataset 3: Estimated population size in large cages.

Code availability statement
The mathematical algorithm that is deemed central to the conclusions is available in supplementary methods. The simulation codes are available from Github: https://github.com/AceRNorth/TerniLargeCage.

Field-specific reporting

Please select the one below that is the best fit for your research. If you are not sure, read the appropriate sections before making your selection.

☐ Life sciences ☐ Behavioural & social sciences ☒ Ecological, evolutionary & environmental sciences

For a reference copy of the document with all sections, see nature.com/documents/nr-reporting-summary-flat.pdf

Ecological, evolutionary & environmental sciences study design

All studies must disclose on these points even when the disclosure is negative.

Study description	Initially, we assessed life history traits of both Ag(QFS1) males and females as well as of the wild-type strain G3 of Anopheles gambiae and assessed their longevity under large cage conditions (4.7 m3) in order to emulate more natural population dynamics (Pollegioni et al. 2020) (see Fig. 1, Supplement Material). Considering the initial Kaplan-Meier Survival estimate of wild-type G3 adult mosquitoes in 4.7 m3 cages of 2m x 1m x 2.35m size and the establishment of overlapping generations with bi-weekly introductions of 400 G3 pupae with a start-up population of 800 mosquitoes, we then analysed age-structured large cage (ASL) populations with an expected mean size of ~570 adult mosquitoes as 'receiving' populations for gene drive release experiments (Supp. Data 3). To mimic field-like conditions absent in small cage conditions, the climate chambers were maintained under near-natural environmental conditions including simulated dusk, dawn and daylight, and each cage was equipped with proven swarming stimuli and a resting shelter (Facchinelli et al. 2015) (Fig. 1). Under these conditions male swarming, an important component of successful mating behaviour, was frequently observed. To mimic a hypothetical field gene drive release, we seeded Ag(QFS1) mosquitoes over a single week (two releases) into the established 'receiving' wild-type populations at two different starting frequencies, low (12.5% initial allele frequency) and medium (25% allele frequency), as well as control cages (0% gene drive release), all in duplicate (6 cages total). The ASL population dynamics and the potential selection of drive-resistant alleles were monitored in treated and control cages until wild-type populations were fully suppressed by the gene drive in the treatments. Finally, we constructed an individual-based stochastic simulation model of the experiment to better understand the observed dynamics of the gene drive frequency and population suppression.
Research sample	Two Anopheles gambiae mosquito strains were used, the wild-type G3 strain (MRA-112) and Female Sterile Gene Drive strain, Ag (QFS)1, previously known as dsxFCRISPRh which contains the gene drive construct inserted in the genetic background of the G3 strain (Kyrou et al. 2018).
Sampling strategy	The gene-drive release to indoor contained ASL populations was performed at two different release frequencies, and each duplicated. Key indicators of population fitness and drive invasion were monitored for the duration of the experiment, including total egg output, hatching rate, pupal mortality, and the frequency of transgenics amongst L1 offspring and the pupal cohorts used for restocking. Total larvae were counted and screened for RFP fluorescence linked to Ag(QFS)1 using the COPAS larval sorter, and 1000 randomly selected to rear at a density of 200 per tray. Pupae positive for the gene drive element could be identified by expression of the RFP marker gene that is contained within the genetic element. Triplicate samples of up to 400 L1 larvae were stored in absolute ethanol at -80°C for subsequent analysis. To further investigate resistance in the large caged release experiment, we analysed mutations found at the genomic target of Ag(QFS)1 in samples collected at early and late timepoints. Genomic DNA (gDNA) was extracted en masse from triplicate samples of 400 L1 larvae, or 50-300 larvae where larval numbers were limiting, that were collected after blood meals given on days 4 and 193 from all 6 cages, and on day 235 where sufficient larvae were available.
Data collection	Life history parameters:<br>To assess life history parameters of wild-type G3 and Ag(QFS)1 strains, standardized phenotypic assays were performed as described in Pollegioni et al. (2020). In brief, clutch size, hatching rate, larval, pupal and adult mortality rates, as well as the bias in transgenics among the offspring of heterozygous Ag(QFS)1 were measured in wild-type G3 and Ag(QFS)1 strains in triplicate in standard small laboratory cages (BugDorm-4). Ag(QFS)1 heterozygotes used in these assays had inherited the drive allele paternally and were therefore subject to paternal, but not maternal, effects of embryonic nuclease deposition that can lead to a mosaicism of somatic mutations at the doublesex locus and a resultant effect on fitness (Kyrou et al. 2018). 150 females and 150 males were mated to wild-type mosquitoes for 4 days, blood-fed, and their progeny counted as eggs using EggCounter v1.0 software (Mollahosseini et al. 2012). Hatching rate was evaluated 3 days post oviposition by visually inspecting 200 eggs under a stereomicroscope (Stereo Microscope M60, Leica Microsystems, Germany). Sex-specific larval mortality was calculated by rearing 200 larvae/tray and counting/sexing the number of surviving pupae. Sex-specific adult survival was assessed in triplicate for each genotype separately by introducing and sexing 100 male and 100 female pupae of G3 and heterozygous Ag(QFS)1 into either small (0.0049 m^3) or large cages (4.7 m^3) (Supp. Fig. 3). In the small cages, we tested 100 individuals in each cage divided by genotype and sex. In each large cage, 100 males and 100 female pupae following sexing and counting were tested together. Because homozygous Ag(QFS)1 do not show clear

sex-specific phenotypes as pupae (Kyrou et al. 2018), 100 Ag(QFS)1 total homozygotes (males and intersex females) were introduced into the small and large cages unsexed (Supp. Fig. 3a). Sex-specific survival of emerged adults was calculated from daily collections of dead adult mosquitoes from the respective cages and their sexing. The adult survival assays in large cages were performed twice, one before the large cage Ag(QFS)1 release experiment started and one after the large cage Ag(QFS)1 release experiment finished. For the latter adult survival assay, around 400 individual mosquitoes were collected from large cage populations at larval stage (before the cage populations declined, day 231 and 311 post-release for Ag(QFS)1 and G3 wild type, respectively), and kept in small cages until the start of the assay (Supp. Fig. 3b).

Large cage data:
Key indicators of population fitness and drive invasion were monitored for the duration of the experiment (2x per week), including total egg output, hatching rate, pupal mortality, and the frequency of transgenics amongst L1 offspring and the pupal cohorts used for restocking. Total larvae were counted and screened for RFP fluorescence linked to Ag(QFS)1 using the COPAS larval sorter, and 1000 randomly selected to rear at a density of 200 per tray. Pupae positive for the gene drive element could be identified by expression of the RFP marker gene that is contained within the genetic element. Triplicate samples of up to 400 L1 larvae were stored in absolute ethanol at -80°C for subsequent analysis.

Pooled Amplicon Sequencing data:
gDNA extractions were performed using the DNeasy Blood & Tissue kit (Qiagen). 100 ng of extracted gDNA was used to amplify a 291 bp region spanning the target site of Ag(QFS)1 in doublesex, using the KAPA HiFi HotStart Ready Mix PCR kit (Kapa Biosystems) and primers containing Illumina Genewiz AmpEZ partial adaptors. PCR reactions were performed under non-saturating conditions and run for 25 cycles, as in Hammond et al. (2017), to maintain proportional representation of alleles from the extracted gDNA in the PCR products. Pooled amplicon sequencing reads, averaging approximately 1.5 million per condition, were analysed using CRISPResso2 (Clement et al. 2019), using an average read quality threshold of 30. Insertions and deletions were included if they altered a window of 20 bp surrounding the cleavage site that was chosen on the basis of previously observed mutations at this locus (Kyrou et al. 2018). Individual allele frequencies were calculated based upon their total frequency in triplicate samples. A threshold frequency of 0.25% per mutant allele was set to distinguish putative resistant alleles from sequencing error (Pfeiffer et al. 2018).

Modelling:
A stochastic model was set up to replicate the experimental design with respect to twice-weekly egg-laying, the initiation phase, the transgene introductions, and the subsequent monitoring phase (Supp. Methods). In brief, daily changes to the population result from egg laying, deaths, and matings, and are assumed to occur with probabilities that may be genotype specific. Adult longevity parameters were estimated from the large cage survival assays that were performed before the gene-drive release experiments began, and after the gene-drive dynamics had run their course. The ASL caged populations showed a similar trend of increasing egg output over time prior to the suppressive effect of the drive (Fig. 2a-c) that may be explained by a general increase in adult survival that was observed between the start and end of the population experiment (Supp. Fig. 3). To account for these changes in the stochastic model, we assumed a small increase in adult survival over time, irrespective of genotype, based on experimental data (Supp. Fig 3). We were particularly interested in the drive allele fertility costs, because these are potentially important to drive allele dynamics in natural populations (Beaghton et al. 2019, North et al. 2020). Fertility costs may arise from paternal and maternal effects of Cas9 deposition into the sperm or egg, or from ectopic activity of Cas9 in the soma (Kyrou et al. 2018). It is therefore possible that female offspring of transgenic fathers differ, in terms of fertility, from female offspring of transgenic mothers, and to investigate this possibility we fitted a separate parameter for the fertility of each type of female. We compared the data to model simulations using a suite of summary statistics (Csilléry et al. 2010; Supp. Methods) to infer the fertility of females with a transgenic father or mother. In addition, we inferred two parameters that determined the egg production of unaffected (wildtype) females, and one parameter that determined the rate of R2 allele creation. We obtained a posterior distribution for all five parameters by retaining the 200 best fitting parameter combinations from 50,000 parameter samples generated by a Monte-Carlo algorithm (Supp. Table 1).

Phenotypic data and DNA samples for Pooled amplicon sequencing were collected by PP, TP, RMi, AT, AB, AS and RMu. The data of pooled amplicon sequencing processed by IM, KK, AH, and TN. The modelling has been performed by AN. Conceptualization: AH, PP, TN, RMu, AC; Methodology: AH, PP, TP, AN, AS, RMu, TN, AC; Investigation: PP, TP, AC, RMi, AT, AB, KK, IM, AS, and RMu; Formal analysis AH, PP, TP, IM, AN, AS, TN and RMu.

Timing and spatial scale

The life-history data of Ag(QFS)1 were collected between 02/2018 to 02/2020.
To assess invasion dynamics of the Ag(QFS)1 strain in ASL populations of An. gambiae, we performed duplicate releases designed to randomly seed ASL populations at low (12.5%, cages 2 & 5) or medium (25%, cages 3 & 6) allelic frequencies. After 74 days pre-release initiation period (22/11/2018-03/02/2019), heterozygous Ag(QFS)1 males were released into duplicate cages in addition to the regular re-stocking of the ASL populations with wild-type pupae (1st release at 04/02/2019; 2nd release at 07/02/2019). Releases took place on two consecutive restocking occasions, representing 15.2% (71 & 72) or 26.3% (142 & 143) of pupae introduced that week (943 and 1085, respectively), equivalent to 25% or 50% of the estimated mean pre-released adult population (on average 574 mosquitoes were present in large cages). No further releases were carried out and indoor ASL populations were maintained through restocking of 400 pupae twice per week. From then, the ASL populations were maintained in the same way we established the receiving population, with the same constant re-stocking rate from offspring. Age-structured mosquito populations were re-stocked twice-weekly through the addition of 400 mosquito pupae (non-sexed). No adult mosquitoes were removed from the cages. Duplicate control cages were similarly maintained, but without release of Ag(QFS)1. Six cages were used in total, each with volume of 4.7 m3 and dimensions of 2 m x 1 m x 2.35 m (length, width, height). The experiment ended at 23/12/2019.
The genomic DNA from large-cage populations was extracted in October 2020, the Pooled amplicon sequencing took place in November 2020, and the respective data processing in February 2021.

Data exclusions

No data were excluded.

Reproducibility

All attempts at replication were successful. Large cage invasion experiments were performed at two release frequencies, and each performed in duplicate. Two control cages (wild type mosquitoes only) were also monitored for comparison. For sampling of target site variation through amplicon sequencing, samples of 1,000 randomly selected L1 larvae were removed in triplicate from each cage, twice-monthly.

Randomization

Experimental groups in lifecycle experiments were randomly distributed. To control for random effects that could affect reproductive

Randomization	capacity of the ASL populations independently of the effect of the gene drive, we chose as control populations those cages with reproductive output at the upper and lower end of the distribution (cages 1 & 4). Replicate gene-drive release cages were distributed to cages 2 and 5 (12.5% allelic frequency) and cages 3 and 6 (25% allelic frequency) to mitigate against potential local environmental position effects (Fig. 1). All subsequent sampling from the large cages described in the paper was performed randomly.
Blinding	Blinding was not possible in this study given the fluorescence marker assessed in ASL populations.

Did the study involve field work? ☐ Yes ☒ No

Reporting for specific materials, systems and methods

We require information from authors about some types of materials, experimental systems and methods used in many studies. Here, indicate whether each material, system or method listed is relevant to your study. If you are not sure if a list item applies to your research, read the appropriate section before selecting a response.

Materials & experimental systems

n/a	Involved in the study	
☒	☐	Antibodies
☒	☐	Eukaryotic cell lines
☒	☐	Palaeontology and archaeology
☐	☒	Animals and other organisms
☒	☐	Human research participants
☒	☐	Clinical data
☒	☐	Dual use research of concern

Methods

n/a	Involved in the study	
☒	☐	ChIP-seq
☐	☒	Flow cytometry
☒	☐	MRI-based neuroimaging

Animals and other organisms

Policy information about studies involving animals; ARRIVE guidelines recommended for reporting animal research

Laboratory animals	The study did not involve vertebrate animals, but invertebrates: Two Anopheles gambiae mosquito strains were used, the wild-type G3 strain (MRA-112) and Female Sterile Gene Drive strain, Ag(QFS)1, previously known as dsxFCRISPRh (Kyrou et al. 2018).
Wild animals	The study did not involve wild animals.
Field-collected samples	The study did not involve samples collected in the field.
Ethics oversight	No ethical approval was required for this study. Anopheles gambiae mosquito strains were contained in a purpose-built and certified Arthropod Containment Level 2 plus facility at Polo d'Innovazione di Genomica, Genetica e Biologia, Genetics & Ecology Research Centre, Terni, Italy.

Note that full information on the approval of the study protocol must also be provided in the manuscript.

Flow Cytometry

Plots

Confirm that:

☒ The axis labels state the marker and fluorochrome used (e.g. CD4-FITC).

☐ The axis scales are clearly visible. Include numbers along axes only for bottom left plot of group (a 'group' is an analysis of identical markers).

☐ All plots are contour plots with outliers or pseudocolor plots.

☐ A numerical value for number of cells or percentage (with statistics) is provided.

Methodology

Sample preparation	L1 larvae were screened for fluorescent markers en masse.
Instrument	Complex Object Parametric Analyzer and Sorter
Software	COPAS, Union Biometrica, Boston, USA
Cell population abundance	L1 larvae were screened for fluorescent markers en masse.
Gating strategy	Is not applicable. Flow cytometry was not used to investigate cells.

☐ Tick this box to confirm that a figure exemplifying the gating strategy is provided in the Supplementary Information.
